# Crosstalk between CXCL12/CXCR4/ACKR3 and the STAT3 Pathway

**DOI:** 10.3390/cells13121027

**Published:** 2024-06-13

**Authors:** Zelong Ma, Faxiao Zhou, Hua Jin, Xiaoming Wu

**Affiliations:** Laboratory of Molecular Genetics of Aging & Tumor, Medical School, Kunming University of Science and Technology, Chenggong Campus, 727 South Jingming Road, Kunming 650500, China; mazeloong@163.com (Z.M.); 13765899802@163.com (F.Z.); 17387633319@163.com (H.J.)

**Keywords:** CXCL12/CXCR4/ACKR3 axis, STAT3 signaling pathway, targeted therapy, JAK2, IL6

## Abstract

The reciprocal modulation between the CXCL12/CXCR4/ACKR3 axis and the STAT3 signaling pathway plays a crucial role in the progression of various diseases and neoplasms. Activation of the CXCL12/CXCR4/ACKR3 axis triggers the STAT3 pathway through multiple mechanisms, while the STAT3 pathway also regulates the expression of CXCL12. This review offers a thorough and systematic analysis of the reciprocal regulatory mechanisms between the CXCL12/CXCR4/ACKR3 signaling axis and the STAT3 signaling pathway in the context of diseases, particularly tumors. It explores the potential clinical applications in tumor treatment, highlighting possible therapeutic targets and novel strategies for targeted tumor therapy.

## 1. Introduction

Crosstalk between the CXCL12/CXCR4/ACKR3 axis and the STAT3 signaling pathway has been hypothesized on the basis of findings that both the CXCL12/CXCR4/ACKR3 axis and STAT3 signaling pathway are involved in the progression of several cancers. In addition, it has been suggested that crosstalk between the CXCL12/CXCR4/ACKR3 axis and the STAT3 signaling pathway plays a role in other systems.

In the following sections, we will introduce the CXCL12/CXCR4/ACKR3 Axis and the STAT3 signaling pathway, followed by a summary of suggested mechanisms leading to observed CXCL12/CXCR4/ACKR3 Axis and STAT3 pathway crosstalk. We will discuss targeting opportunities for simultaneous regulation of the CXCL12/CXCR4/ACKR3 Axis and STAT3 pathway in therapeutic development.

## 2. Background

### 2.1. The CXCL12/CXCR4/ACKR3 Axis

CXCL12, also referred to as stromal cell-derived factor-1 (SDF-1) or pre-B cell growth-stimulating factor (PBSF), is a member of the CXC chemokine subfamily. Current research indicates that CXCL12 possesses two specific receptors, namely, CXCR4 and ACKR3. CXCL12 is the only recognized CXC chemokine that undergoes differential mRNA splicing [1]. As a result of its selective gene splicing, seven isoforms of CXCL12 have been identified: α, β, γ, δ, ε, θ, and iso7. Among these isoforms, α and β are the most prevalent and have been extensively studied [2]. The seven isoforms are encoded by the same CXCL12 gene and share the first three exons. Their distinction lies in the fourth exon, which determines the length of the splice variants. In fact, all CXCL12 isoforms share the initial 67 amino acids, albeit varying in length [3]. CXCL12α, composed of 68 amino acids, is expressed in various tissues, with a significant enrichment in stromal cells and endothelial cells. It participates in a wide range of physiological and pathological processes. In contrast, CXCL12β, which contains an additional four amino acids compared to CXCL12α, exhibits similar functional properties. However, the β isoform possesses enhanced angiogenic potential and is predominantly enriched in vascular-rich organs such as the kidney, liver, and spleen [4,5]. The remaining splice variants of CXCL12 are distinguished by differences in their fourth exon [3]. CXCL12γ is primarily found in metabolically active organs with low vascularization, such as the brain and heart. Notably, the γ isoform exhibits the highest affinity for its receptor, CXCR4, and exerts the longest-lasting downstream effects [6].

The C-X-C chemokine receptor 4 (CXCR4), also known as CD184, is a member of the seven-transmembrane G protein-coupled receptor family. CXCR4 is a chemokine receptor akin to rhodopsin, characterized by seven transmembrane domains and typically classified as a G protein-coupled receptor (GPCR) [7]. CXCL12 binds to the extracellular domains of CXCR4, leading to the conversion of GDP-bound G proteins to their GTP-bound forms. This conversion results in the dissociation of heterotrimeric G proteins into Gα subunits and Gβγ subunits. The released Gα and Gβγ subunits subsequently activate downstream molecules, including protein kinase B (AKT), c-Jun N-terminal kinase (JNK), mitogen-activated protein kinase (MEK), and extracellular signal-regulated kinase 1/2 (ERK1/2), thereby modulating transcription [8]. In addition, the CXCL12/CXCR4 signaling axis can activate the Janus kinases/signal transducers and activators of transcription (JAK/STAT) pathways [9], the Wnt/β-catenin signaling pathway [10], and the Ca2+/calmodulin-dependent protein kinase II/AMP response element-binding protein (CaMKII/CREB) signaling pathway [11]. However, the specific G protein subunit through which CXCR4 activates these pathways remains an area of active investigation. Based on the coupling Gα subunits, diverse GPCR signaling pathways can be classified into four families: Gα_s_, Gα_i_, Gα_q_, and Gα_12_ [12]. Initially, CXCR4 was categorized as a Gα_i_ protein-coupled receptor [13]. Upon the binding of CXCL12 to CXCR4, the G protein-coupled receptor (GPCR) is activated. The activation of the Gα_i_ subunit inhibits adenylyl cyclase, which catalyzes the conversion of 5′-triphosphate adenosine (ATP) to cyclic adenosine monophosphate (cAMP). The cAMP molecules bind to the two cAMP-binding domains in the regulatory subunit of protein kinase A (PKA), leading to the release of PKA’s two catalytic subunits. Consequently, downstream target proteins are activated and phosphorylated [14,15].

ACKR3, also known as CXCR7 or RDC1, is a member of the seven-transmembrane G protein-coupled receptor (GPCR) family, akin to CXCR4. Unlike CXCR4, ACKR3 is an atypical G protein-coupled receptor. Despite ACKR3 harboring two amino acid substitutions within the prototypical DRYLAIV motif for G protein interaction, the reconstitution of this motif does not enable ACKR3 to signal via G proteins. Consequently, ACKR3 demonstrates a reduced affinity for G proteins, while it exhibits an elevated affinity for the scaffold protein β-arrestin2 (ARRB2) [16,17]. As a result, upon binding to its ligand CXCL12, ACKR3 does not dissociate from G proteins. Instead, it activates the G protein-coupled receptor kinase 2 (GRK2) and undergoes receptor phosphorylation, leading to conformational changes. Subsequently, ARRB2 is recruited to activate downstream signaling pathways. Notably, studies have shown that in a mouse model with CXCR4 knockout, ACKR3-positive neural progenitor cells exhibit migration along the CXCL12 gradient in vitro [18]. Moreover, the inhibition of ACKR3 suppresses CXCL12-induced migration of cervical cancer cells, whereas CXCR4 does not participate in these processes [19]. Therefore, the signaling pathways mediated by ACKR3 may diverge from the canonical chemokine pathways governed by CXCR4. By binding to CXCR4/ACKR3 through autocrine or paracrine mechanisms, CXCL12 activates downstream signaling pathways, including STAT3, NFκB, and MAPK. These pathways are instrumental in various biological processes, such as development, homeostasis maintenance, the regulation of inflammatory responses, and the facilitation of malignant tumor progression [20,21,22,23].

### 2.2. The STAT3 Signaling Pathway

The Signal Transducer and Activator of Transcription (STAT) protein family consists of seven members: STAT1, STAT2, STAT3, STAT4, STAT5A, STAT5B, and STAT6 [24]. These proteins can transmit signals from both receptor-associated and non-receptor-associated kinases to the cell nucleus [25]. Among the seven members of the STAT protein family, STAT3 emerges as the most potent promoter of tumor growth and immune evasion [26,27]. Notably, it is the only family member whose genetic deletion results in embryonic lethality [28,29].

STAT3 is a protein composed of 770 amino acids and is characterized by six functionally conserved domains. In its inactive state, STAT3 exists as a monomer within the cytoplasm. Studies have indicated that the presence of STAT3 in the cytoplasm is crucial for maintaining the pluripotency of embryonic stem cells and regulating immune responses [29,30]. The Src homology 2 (SH2) domain is the most conserved structural motif within STAT proteins. Upon tyrosine phosphorylation by Janus kinase (JAK)-mediated growth factor receptors, such as gp130, the SH2 domain of STAT3 recognizes and binds to specific phosphotyrosine motifs, playing a critical role in signal transduction [31]. For instance, phosphorylated STAT3 induces the upregulation of regulatory factors involved in cell proliferation (e.g., Cyclin D1 and MYC) and survival (e.g., BCL-xL and survivin), as well as the expression of proangiogenic factors (e.g., VEGF) and immune-suppressive cytokines (e.g., IL-6) [26,32].

The canonical STAT3 pathway is triggered when cytokines such as interleukin-6 (IL-6) bind to their respective receptors (e.g., gp130/IL-6Rα). This ligand–receptor interaction induces the phosphorylation of receptor-associated kinases, known as Janus kinases (JAKs). The subsequent phosphorylation of JAKs leads to the tyrosine phosphorylation of STAT3 at residue Tyr705, thereby activating the STAT3 pathway. Once activated, STAT3 forms homodimers and dissociates from the receptor, translocating into the cell nucleus. Within the nucleus, it binds to specific promoter DNA sequences, driving gene transcription. The activation of the STAT3 pathway, whether through intrinsic or extrinsic mechanisms, exerts a profound influence on the tumor microenvironment. It promotes tumor proliferation, angiogenesis, and immune evasion and contributes to tumor metastasis through various mechanisms, including epithelial–mesenchymal transition and the upregulation of matrix metalloproteinases [33,34,35,36,37]. Intrinsic mechanisms include JAK2/STAT3 signaling transduction and intracellular STAT3’s direct transcriptional regulation of genes associated with tumor progression, such as those promoting the differentiation and cytotoxic functions of tumor-specific T cells [38]. Extrinsic mechanisms encompass the activation of the STAT3 pathway with ionizing radiation [39] or with cytokines produced by inflammatory cells within the tumor microenvironment [40], thereby facilitating tumor development.

The activation of the STAT3 pathway leads to the upregulation of VEGF and specific matrix metalloproteinases (MMPs). These factors play distinct roles: VEGF fosters angiogenesis, while MMPs contribute to invasive processes that propel tumor metastasis [41]. Additionally, STAT3 forms a positive feedback loop by binding to the IL-6 promoter, resulting in an increase in IL-6 expression [42]. Both VEGF and IL-6 possess immunosuppressive properties, facilitating immune evasion by tumor cells with excessive STAT3 activation [43,44]. Furthermore, the protumorigenic effects of STAT3 may involve the induction of miR-21 and miR-181b-1, which, respectively, inhibit the expression of tumor suppressors PTEN and ubiquitin carboxyl-terminal hydrolase CYLD [45]. Notably, apoptosis-related proteins B-cell lymphoma-2 (Bcl-2) and Bcl-2-associated protein X (Bax) serve as transcriptional targets of the STAT3 pathway [46,47]. Upon STAT3 activation, the inhibition of Bcl-2 and Bax promotes cell survival.

The STAT3 pathway can be activated by several kinases, including the epidermal growth factor receptor (EGFR), vascular endothelial growth factor receptor 2 (VEGFR2), hepatocyte growth factor receptor (HGFR), platelet-derived growth factor receptor (PDGFR), SRC, and ABL. Among these, EGFR, VEGFR2, HGFR, and PDGFR are classified as receptor tyrosine kinases (RTKs) with intrinsic tyrosine kinase activities. These RTKs phosphorylate JAK, thereby facilitating the activation of the STAT3 pathway. Additionally, non-receptor tyrosine kinases (nRTKs) such as Src or ABL can also activate STAT3. At the molecular level, Src or ABL induces tyrosine phosphorylation, leading to STAT3 activation and subsequent translocation into the cell nucleus, where it exerts its regulatory functions on gene transcription [48]. Furthermore, in various tumors, including lung cancer, breast cancer, and colorectal cancer, the activation of the CXCL12/CXCR4/ACKR3 signaling axis has been associated with STAT3 pathway activation [49,50,51,52].

The STAT3 pathway is subject to negative regulation by various cellular factors, including suppressor of cytokine signaling (SOCS), protein inhibitor of activated STAT (PIAS), and protein tyrosine phosphatases (PTPs). Among these, SOCS is a protein family characterized by a central Src homology 2 (SH2) domain. SOCS3 is a member of the SOCS family and acts as a negative feedback loop for the STAT3 signaling pathway. Specifically, SOCS3 interacts with the JAK2 domain (or the intracellular portion of the receptor) to exert its effects [53]. In particular, the kinase inhibitory region of SOCS3 blocks the substrate-binding groove on JAK2, thereby inhibiting its kinase activity and promoting the ubiquitination and degradation of receptor proteins, which in turn suppresses STAT3 phosphorylation [53,54]. Additionally, SOCS can interact with receptor tyrosine kinases (RTKs), thereby disrupting STAT3 recruitment [55,56]. Notably, SOCS does not inhibit the non-receptor tyrosine kinase SRC-mediated activation of the STAT3 signaling pathway [57]. The deletion of SOCS3 leads to STAT3 pathway activation, promoting cell proliferation and increasing the incidence of hepatitis-induced liver cancer [58]. Importantly, the upregulation of SOCS3 inhibits tumor growth and metastasis. Exogenous SOCS3 suppresses the growth of human non-small cell lung cancer cells and enhances their sensitivity to radiotherapy [59].

The protein inhibitor of activated STAT (PIAS) functions as an inhibitor of STAT transcriptional activation. PIAS proteins can inhibit STAT transcription through various mechanisms, including blocking the DNA-binding activity of transcription factors, recruiting transcriptional repressors, and facilitating protein SUMOylation modifications (analogous to ubiquitination) [60]. Among these, PIAS3 specifically interacts with activated STAT3 dimers, preventing their binding to DNA and thereby inhibiting STAT3 transcriptional activity [61]. In glioblastoma tissues, an elevated expression of PIAS3 promotes cell proliferation [62,63]. Furthermore, the upregulation of PIAS3 suppresses cell proliferation and enhances chemosensitivity in various tumors. For instance, curcumin, by upregulating PIAS3 in ovarian and endometrial cancer cells, inhibits STAT3 activation and restrains tumor cell growth [64]. Additionally, the overexpression of PIAS3 inhibits lung cancer cell growth and restores sensitivity to chemotherapy [65,66].

Protein tyrosine phosphatases (PTPs) play a pivotal role in negatively regulating STAT3 activity. Seven types of PTPs have been identified as targeting STAT3: PTPRD, PTPRT, PTPRK, SHP-1, SHP-2, PTPN9, and TC-PTP. PTPRD, PTPRT, and PTPRK directly interact with STAT3, dephosphorylating STAT3 at the Tyr705 site to inhibit STAT3 signal transduction [67,68,69]. SHP-1 and SHP-2 possess a phosphatase domain and two SH2 domains. Through their SH2 domains, they interact with target proteins (such as JAK/STAT3, Src family kinases, and growth factor receptors), dephosphorylating substrates via their phosphatase activity [70,71]. In colorectal cancer, the p53R248Q mutation competitively binds to SHP2, leading to STAT3 activation [72]. In 2012, PTPN9 was first reported to induce STAT3 dephosphorylation, suppressing breast cancer growth [73]. Additionally, STAT3 is one of the substrates for TC-PTP. Deficiency of TC-PTP in triple-negative primary breast cancer cells enhances cell proliferation through STAT3 signaling activation and increased Src family kinase activity [74]. Overexpression of TC-PTP significantly inhibits in vitro cell proliferation and suppresses xenograft growth in human breast cancer cell lines [74].

The interaction between the CXCL12/CXCR4/ACKR3 axis and the STAT3 signaling pathway is crucial in the development and progression of various diseases, including cancer. In breast cancer, the CXCL12/CXCR4/ACKR3 axis promoted breast cancer metastasis through the activation of the STAT3 pathway [52,75]. In skin-related disorders, CXCL12 activated the STAT3 pathway, inhibiting hair growth [76]. Additionally, the STAT3 pathway regulated the expression of CXCL12. Understanding the relationship between the CXCL12/CXCR4/ACKR3 axis and the STAT3 pathway could lead to new therapeutic targets for innovative cancer treatments.

## 3. Crosstalk between CXCL12/CXCR4/ACKR3 Signaling Axis and STAT3 Pathway

The CXCL12/CXCR4/ACKR3 signaling axis and the STAT3 pathway are intimately linked with immune regulation and the malignant progression of a variety of diseases, encompassing skin disorders, brain injuries, myocardial damage, and tumors [24,77]. Within this context, the CXCL12/CXCR4/ACKR3 axis exerts both positive and negative effects through the activation of STAT3. Moreover, the activated STAT3 pathway can augment CXCL12 expression, playing a pivotal role in the progression of these diseases. Understanding the relationship between the CXCL12/CXCR4/ACKR3 axis and the STAT3 pathway could lead to new therapeutic targets for innovative cancer treatments

### 3.1. CXCL12/CXCR4/ACKR3 and STAT3 Pathway Crosstalk in Cancer

Research findings indicate that the CXCL12 and STAT3 proteins are significantly upregulated in various malignancies, including breast cancer, lung cancer, hepatocellular carcinoma, esophageal cancer, bladder cancer, and leukemia [78,79,80]. Notably, tumor cells positive for CXCR4 migrate along the CXCL12 concentration gradient to distant organs exhibiting high CXCL12 expression, thereby facilitating tumor metastasis [81]. In bladder cancer tissues, a positive correlation exists between elevated CXCR4 expression and positive p-STAT3 expression [82]. These findings suggest an activating interplay between the CXCL12/CXCR4 signaling axis and the STAT3 pathway.

The CXCL12/CXCR4/ACKR3 signaling axis activates the STAT3 pathway, playing a central role in tumor proliferation, metastasis, anti-apoptosis, maintenance of tumor stemness, and immune escape. In breast cancer tissues, dual positivity for CXCR4 and phosphorylated STAT3 (p-STAT3) correlates positively with tumor size, lymph node metastasis, and histological grade [52]. Further investigations reveal that the CXCL12/CXCR4 axis phosphorylates JAK2, thereby activating the STAT3 signaling pathway to promote breast cancer proliferation and metastasis. Inhibition of either CXCR4 or JAK2 suppresses STAT3 phosphorylation, reversing the proliferative and metastatic phenotypes of breast cancer [52]. Using a mouse orthotopic tumor model constructed with 4T1.2 breast cancer cells, it was observed that 4T1.2 cells can metastasize to the lungs. Cell experiments demonstrated that CXCL12 upregulates STAT3 phosphorylation, while knocking down ACKR3 attenuates the upregulation of p-STAT3^Y705^. These findings suggest that the CXCL12/ACKR3 axis activates the STAT3 signaling pathway. Immunohistochemical analysis of the in situ tumors revealed concurrent elevation of p-STAT3 and the vascular marker CD31, as well as the proliferation marker Ki67, promoting tumor metastasis to the lungs [75].

Epithelial–mesenchymal transition (EMT) plays a pivotal role in tumor metastasis [83]. Furthermore, the enhancement of a tumor’s mesenchymal phenotype is associated with chemotherapy resistance and unfavorable outcomes [84]. In esophageal cancer, in vitro experiments have demonstrated that the CXCL12/ACKR3 axis activates the STAT3 pathway. This activation leads to the downregulation of the epithelial cell marker protein E-Cadherin and the upregulation of mesenchymal cell markers N-Cadherin, Vimentin, Snail, and Slug. Consequently, this process promotes EMT and accelerates the metastasis of esophageal cancer. In vivo tumor transplantation experiments have also revealed that the upregulation of ACKR3 enhances the expression of Ki67 in tumor tissues, thereby promoting the growth and metastasis of esophageal cancer [49]. Similarly, in gastric cancer, activation of the CXCL12/CXCR4 axis via the STAT3 pathway induces downregulation of E-Cadherin and upregulation of N-Cadherin, Vimentin, and Snail, enhancing EMT, migration, and the invasiveness of gastric cancer cells [85]. Additionally, Yang et al. found that downregulation of ACKR3 in acute T-cell lymphoblastic leukemia inhibits STAT3 pathway activation, suppressing tumor growth and lung metastasis in vivo [86]. The silencing of ACKR3 also leads to decreased matrix metalloproteinase-2 (MMP2) and vascular endothelial growth factor (VEGF) expression, inhibiting migration and invasion of tumor endothelial cells in hepatocellular carcinoma [87]. Furthermore, in gastric cancer, ACKR3 has been implicated in promoting tumor growth and metastasis via the STAT3/c-Myc pathway [88].

Tumor stem cells (CSCs) constitute a critical cellular subset in the heterogeneous development of tumors, playing pivotal roles in tumor metastasis, recurrence, and drug resistance [89]. Hong et al. demonstrated that activation of the CXCL12/CXCR4 axis induces immortalized embryonic kidney cells to transform into cells resembling tumor stem cells, potentially contributing to carcinogenesis [90]. In hepatocellular carcinoma, the activation of the CXCL12/CXCR4 axis via the STAT3 signaling pathway promotes protein expression of CSC markers SOX2 and CD133, enhancing the spheroid-forming capacity and invasiveness of liver cancer cells [91].

Additionally, resistance to apoptosis in tumors promotes proliferation and metastasis. Ahn et al. proposed that 3-formylchromone downregulates p-STAT3, attenuating the CXCL12-driven migration and invasiveness of liver cancer cells. Simultaneously, 3-formylchromone inhibits the expression of Bcl-2, Bcl-xL, Survivin, and Mcl-1 in liver cancer cells, suggesting that 3-formylchromone promotes apoptosis in liver cancer cells by suppressing the STAT3 pathway and weakening CXCL12-driven metastasis [70]. In non-small cell lung cancer, in vitro experiments demonstrate that CXCL12 binding to CXCR4, via activation of the JAK2/STAT3 signaling pathway, reduces the proportion of lung cancer cell apoptosis induced by the chemotherapeutic drug cisplatin. Clinical lung cancer tissue samples also reveal a positive correlation between elevated CXCL12 and positive p-STAT3 expression and the malignant progression of lung cancer [50].

Autophagy is a fundamental cellular process that maintains homeostasis by degrading cellular components through lysosomal machinery. It serves as a critical mechanism for supplying cells with essential biomolecules and energy during periods of stress. Selective autophagy is mediated by autophagic receptors, with p62 being one of the earliest identified autophagic receptor proteins. Notably, p62 is closely associated with tumor development [92]. In the context of bladder cancer, cancer-associated fibroblasts (CAFs) secrete CXCL12, which activates the JAK2/STAT3 signaling pathway, leading to the accumulation of p62. This, in turn, promotes the migration and invasion of bladder cancer cells and upregulates the expression of programmed cell death ligand 1 (PDL-1) via non-degradative pathways [93]. Further investigations reveal that CXCL12 secreted by CAFs, upon binding to its specific receptor CXCR4, activates JAK2/STAT3 signaling, resulting in increased expression of cylindromatosis (CYLD), a deubiquitinase. CYLD deubiquitinates p62, leading to its accumulation and the subsequent inhibition of PDL-1 degradation via autophagy pathways [93]. Elevated PD-L1 expression can facilitate immune escape by tumor cells [94,95].

Tumor immune escape, which encompasses both immune suppression and immune resistance, represents a key malignant phenotype of tumor cells. CXCL12, secreted by cancer-associated fibroblasts (CAFs), activates the STAT3 pathway, inducing the generation and activation of myeloid-derived suppressor cells, resulting in tumor immune escape and further promoting tumor development [96]. Multiple studies have demonstrated that within leukemia, the CXCL12/CXCR4 axis plays a pivotal role in maintaining the interactions between leukemia cells and the bone marrow niche. This axis facilitates the adhesion of leukemia cells to the bone marrow, enhancing cell survival and reducing sensitivity to signal transduction inhibitors and chemotherapeutic agents [97,98,99,100]. Additionally, Madrazo E et al. discovered that CXCL12 promotes H3K9 methylation in primary T-cell acute lymphoblastic leukemia (T-ALL) cells, a process occurring within minutes. This suggests that CXCL12-mediated H3K9 methylation alters the overall chromatin conformation of T-ALL cells, enhancing their nuclear deformation and migratory capabilities [101]. The CXCL12/CXCR4/STAT3 pathway can promote the expression of IL-10 in chronic lymphocytic leukemia (CLL) cells, inducing immunosuppression. Treatment with lenalidomide, an immunomodulatory anticancer drug, inhibits STAT3^Y705^ phosphorylation induced with IL-10 in healthy T cells, reversing the T-cell dysfunction induced with CLL [102], indicating the significance of CXCL12/CXCR4 in leukemia treatment. Concurrently, research by Sison EA et al. has shown that chemotherapy for acute lymphoblastic leukemia (ALL) upregulates CXCR4 expression in all cells. Co-culturing ALL cells with bone marrow stromal cells (capable of producing CXCL12) generates drug resistance and protects ALL cells from chemotherapy-induced apoptosis, leading to further exacerbation of leukemia [103]. In summary, various studies suggest that CXCR4 inhibition may be beneficial for the treatment of ALL, yet further research is required to fully understand the comprehensive effects of targeting the leukemia microenvironment.

### 3.2. CXCL12/CXCR4/ACKR3 and STAT3 Pathway Crosstalk in Other Diseases

The CXCL12/CXCR4/ACKR3 signaling axis and the STAT3 pathway play crucial roles in inflammation and autoimmune diseases. In various diseases, the CXCL12/STAT3 signaling cascade exhibits diverse functions, both beneficial and malignant. For instance, in Toxoplasma-induced brain injury, elevated mRNA expression of CXCL12 and STAT3, in conjunction with other molecules, orchestrates innate immunity and T cell-mediated protective responses, effectively controlling cerebral Toxoplasma infection [104]. Additionally, cardiac stem cells, through paracrine secretion of CXCL12 and activation of the STAT3 pathway, ameliorate acute myocardial function following ischemia [105]. These stem cells contribute to post-ischemic myocardial recovery by mobilizing to injured tissue and enhancing local vascular regeneration after myocardial infarction, thereby mediating cardiac protection [106]. In cardiac myocytes, the miR-135a-5p/CXCL12/JAK2-STAT signaling axis promotes post-myocardial infarction inflammation and apoptosis, ultimately mitigating the extent of myocardial injury [107]. Furthermore, in the context of local vascular injury following arterial damage, the JAK/STAT signaling pathway induces the upregulation of vascular CXCL12 expression, promoting proliferative responses during vascular remodeling [108].

CXCL12 is also highly expressed in dermal cells, and during the process of wound healing, overexpressed IL-10 activates the STAT3 pathway, promoting fibroblast-specific hyaluronic acid synthesis and facilitating tissue regeneration. Furthermore, IL-10 enhances the expression of CXCL12 and VEGF in fibroblasts [109,110]. In studies related to skin-associated diseases, the activation of CXCR4 by CXCL12 was found to promote the phosphorylation of STAT3 and STAT5 in the dermal papilla layer and outer root sheath cells, thereby inhibiting hair growth [76].

Zhuang et al. discovered that in *Helicobacter pylori*-associated gastritis, the E-box basic helix–loop–helix protein 40 (BHLHE40) translocates to the cell nucleus and interacts with p-STAT3 (Tyr705), which is activated by the cagA protein. This interaction results in an upregulation of CXCL12 expression. The CXCL12, released by gastric epithelial cells, promotes CD4+ T cell infiltration in the gastric mucosa [111]. BHLHE40, a member of the basic helix–loop–helix transcription factor family, has been reported to play a crucial role in inflammatory diseases. Notably, in *H. pylori*-infected vascular endothelial cells, BHLHE40 expression is induced via the CagA/ERK pathway [111].

In a study conducted in 2020 on renal fibrosis, it was found that the binding of CXCL12α to CXCR4 instigates the phosphorylation of JAK2 and JAK3. This event triggers the activation of STAT3 and STAT6. The activation of these proteins subsequently inhibits the transcription of GSK3β and facilitates the assembly of the β-catenin degradation complex. As a result, β-catenin is liberated from the cytoplasmic degradation complex, leading to its accumulation in the nucleus. The nuclear β-catenin then mediates renal tubular cell fibrosis by interacting with the transcription factor TCF/LEF [112]. In a related study, Wang et al. provided evidence that in the context of diabetic kidney injury, the CXCL12α/CXCR4/STAT3 signaling pathway mediates the upregulation of DPP4-induced mitochondrial dysfunction in renal tubular cells. This finding underscores the critical role of this signaling pathway in renal pathology [113].

### 3.3. Mechanism of STAT3 Pathway Activation by CXCL12/CXCR4/ACKR3 Axis

**Figure 1:**
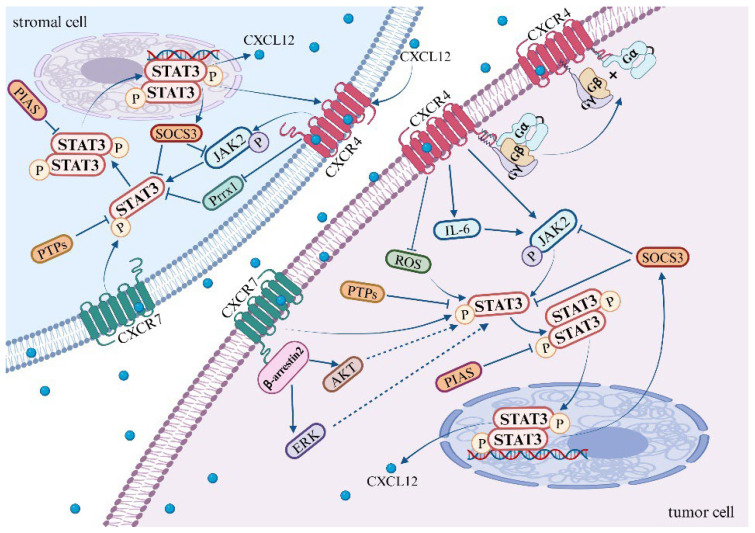
Mechanisms of the STAT3 pathway activation by CXCL12/CXCR4/ACKR3 axis.

In 1999, it was initially observed that the dimerization of CXCR4 is induced upon its binding to its ligand CXCL12, which in turn activates the JAK/STAT signaling pathway [114]. Further investigations have unveiled that the activation of JAK2 is a result of CXCL12 binding to CXCR4, which subsequently leads to the activation of the STAT3 signaling pathway [9,115] (Figure 1). Ahr et al. provided compelling evidence that two distinct structural domains of CXCR4 are involved in JAK2/STAT3 signal transduction. More specifically, the N-terminal portion of the third intracellular loop (ICL3) of CXCR4 is responsible for the activation of JAK2, which results in the phosphorylation of tyrosine at residue 157 (Tyr157) within the C-terminal portion of the second intracellular loop (ICL2). This event ultimately leads to the activation of the STAT3 pathway [116]. This observation underscores the intricate molecular mechanisms involved in signal transduction pathways.

CXCR4 activates the JAK2/STAT3 pathway through the association of heterotrimeric G proteins with GPCRs [117]. The precise mechanistic underpinnings of this association remain to be elucidated. However, Soriano SF et al. discovered that the binding of heterotrimeric G proteins to G protein-coupled receptors (GPCRs) is contingent upon JAK activation. This process involves the upregulation of the suppressor of cytokine signaling (SOCS3), which, by binding to CXCR4, impedes the JAK/STAT and Gi pathways without disrupting the expression of chemokine receptors on the cell surface [115]. Existing research suggests that the activation of CXCL12/CXCR4 leads to an upregulation in the expression of SOCS3 within the STAT3 pathway. Subsequently, SOCS3 binds to CXCR4, providing a negative feedback mechanism that inhibits the STAT3 pathway [55,56,115,118]. It is noteworthy that the JAK2 inhibitor AG490 mitigates the upregulation of SOCS3, whereas the G protein inhibitor pertussis toxin (PTX) does not exert any influence on the expression of SOCS3.

Kim et al. uncovered that the upregulation of CXCR4, when stimulated by AKT and JNK, fosters the expression and secretion of IL-6. Following this, IL-6 activates the JAK/STAT3/Snail signaling axis, which leads to the depletion of the nuclear protein UHRF1, thereby inducing EMT in liver cancer cells [119]. Additionally, CXCR4 may activate the STAT3 pathway by inhibiting the production of reactive oxygen species (ROS). Under hypoxic conditions, ROS are known to mediate the activation of the STAT3 pathway. Notably, in tumor-targeted therapy, both curcumin and a novel dimanganese complex (FMSP) have been observed to downregulate CXCR4, thereby promoting the generation of ROS and inhibiting the activation of the STAT3 pathway [120,121]. Furthermore, the vascular endothelial growth factor (VEGF) may mediate the activation of CXCR4 in the STAT3 pathway. Clinical samples from lung cancer patients have demonstrated the co-expression of CXCR4, p-STAT3, and VEGF-A, suggesting a potential role in tumor progression and angiogenesis in non-small cell lung cancer (NSCLC) [122].

In addition to the aforementioned pathways, several other signaling pathways have been reported to activate the STAT3 pathway. The binding of vascular endothelial growth factor (VEGF) and epidermal growth factor (EGF) to their respective receptors instigates receptor dimerization. The phosphorylation of these dimerized receptors occurs reciprocally, with VEGFR inducing JAK2 phosphorylation and EGFR inducing c-Src kinase phosphorylation, thereby facilitating the activation of the STAT3 pathway [123]. Moreover, mTORC1 has also been found to activate the STAT3 pathway, thereby promoting angiogenesis [124]. Apart from IL-6, other members of the interleukin family, such as IL-10, IL-17, IL-19, and IL-22, are capable of activating the STAT3 pathway [125,126,127,128]. Although there is currently no definitive research demonstrating whether CXCR4 activates STAT3 through these pathways, further investigation may elucidate the specific mechanisms underlying CXCR4-mediated STAT3 activation.

ACKR3, identified in 2005, functions as an additional specific receptor for CXCL12 [129]. Current research has shown that the activation of CXCL12/ACKR3 contributes to the progression of certain diseases and tumors by facilitating STAT3 signaling. However, the exact mechanism through which ACKR3 activates the STAT3 pathway remains to be comprehensively elucidated. In studies pertaining to ACKR3, it has been found that the binding of CXCL12 to ACKR3 recruits β-arrestin2, which subsequently activates the AKT and ERK signaling pathways [8,130]. Notably, the activation of either the AKT or ERK pathway has been linked with enhanced STAT3 transcriptional activity [90,131], providing valuable insights for future investigations into the mechanisms underlying STAT3 pathway activation.

In addition to its interaction with its cognate receptor, CXCL12 has the ability to modulate transcriptional co-activators, thereby promoting the transcriptional activation of STAT3. In the pre-metastatic microenvironment of hepatocellular carcinoma, the expression of CXCL12 can downregulate the transcription factor Paired Related Homeobox 1 (Prrx1), thereby inducing an increase in the protein and mRNA expression of CXCR4 in hepatocellular carcinoma cells via the STAT3 pathway [91]. Specifically, the binding of CXCL12 to CXCR4, under the mediation of Prrx1, promotes the nuclear translocation and phosphorylation level of STAT3, activates the STAT3 signaling pathway, and upregulates the expression of CXCR4. In the pre-metastatic niche, CXCL12 recruits circulating tumor cells with high CXCR4 and low Prrx1 expression from the bloodstream, ultimately promoting the metastatic colonization of distant organs [91].

### 3.4. Mechanism of STAT3 Signaling Pathway Upregulation of CXCL12 Expression

The expression of CXCL12 is also modulated by the STAT3 signaling pathway. Activated STAT3, by upregulating CXCL12, promotes tumor cell migration. Immunosuppressive cytokines (such as IL-10, PD-L1, and TGF-β) activate the STAT3 signaling pathway, leading to tumor immune suppression and epithelial–mesenchymal transition. Consequently, CXCL12 expression is enhanced in primary tumors [132]. In related studies involving fibroblasts, both aging and p53 mutations activate the STAT3 pathway in fibroblasts, leading to increased secretion of CXCL12. This induction of CXCL12 contributes to the acquisition of cancer-associated fibroblast (CAF) characteristics, ultimately promoting tumor growth and metastasis [133,134].

In bone marrow stromal cells, increased expression of heme oxygenase-1 (HO-1) facilitates JAK2/STAT3 phosphorylation, subsequently promoting the secretion of CXCL12 and activation of the CXCL12/CXCR4 signaling pathway [135]. This molecular cascade is implicated in various signal transduction pathways related to cell survival and proliferation. Although several studies suggest that the STAT3 pathway upregulates CXCL12 expression, the precise regulatory mechanisms remain elusive.

In the context of other diseases, it has been found that p-STAT3 binds to the CXCL12 promoter to enhance its expression. Li ZY and colleagues discovered that p-STAT3 can directly bind to a 163-bp fragment of the CXCL12 promoter from −1608 to −1770, promoting the expression of CXCL12 in the dorsal root ganglia (DRG), leading to chronic pain after oxaliplatin treatment [136]. Research by Xin WJ and others showed that long-term morphine treatment increased the binding of p-STAT3 to the specific binding site (−1667/−1685) of the CXCL12 promoter [137] and that p21 inhibited the binding of STAT3 to the CXCL12 promoter [108]. At the same time, p-STAT3 regulates histone modifications in the CXCL12 promoter region. It enhances the interaction between the acetyltransferase p300 and STAT3, inducing histone H4 acetylation in the CXCL12 promoter region, which promotes the expression of CXCL12 [137].

The STAT3 pathway has the capacity to modulate other transcription factors, thereby enhancing CXCL12 expression. Kim et al. revealed that in normal fibroblasts, the activation of the IL-6/STAT3 pathway leads to an upregulation of Twist1. This upregulation subsequently augments CXCL12 expression, thereby facilitating the transformation of normal fibroblasts into cancer-associated fibroblasts (CAFs) [138]. Furthermore, it has been observed that activated STAT3 can induce a phenotypic transition in tumor-associated macrophages (TAMs) from M1 to M2 via NF-κB signaling within the tumor microenvironment (TME). This transition results in immune suppression and the induction of EMT in colorectal cancer, thereby promoting the expression of CXCL12 in primary tumors [132].

## 4. Interplay between the CXCL12/CXCR4/ACKR3 Axis and the STAT3 Pathway in Coordination with Other Signaling Pathways

**Figure 2:**
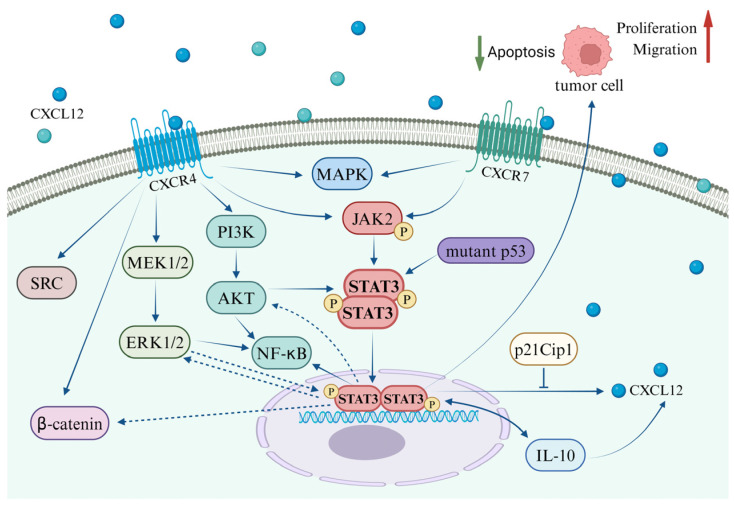
Interplay between the CXCL12/CXCR4/ACKR3 axis and the STAT3 pathway in coordination with other signaling pathways.

Tumor growth and metastasis represent a multifaceted process encompassing multiple stages that involve the regulation of a variety of growth factors and signaling pathways [139]. Upon interaction with its specific receptors, CXCL12, secreted by either stromal cells or tumor cells, has the potential to activate distinct signaling pathways in various environments. These pathways often demonstrate parallel or intersecting relationships (Figure 2). Notably, research on prostate cancer has identified that CXCL12 secreted by stromal cells can stimulate the invasive characteristics of prostate cancer cells, potentially playing a significant role in tumor progression associated with obesity [140]. Further mechanistic investigations suggest that the binding of CXCL12 to its receptors concurrently activates the STAT3, NFκB, and MAPK signaling pathways [140]. However, the reciprocal regulatory interactions among STAT3, NFκB, and MAPK induced by CXCL12 are yet to be fully elucidated. Jin et al. have documented the regulatory effects of the MAPK/STAT3/NFκB signaling cascade in the treatment of gastric cancer cells [141,142].

In a variety of tumor cells, including gastric cancer, glioblastoma, and breast cancer, the activation of the CXCL12/CXCR4 axis has been observed to stimulate the SRC, ERK, and STAT3 pathways. This stimulation promotes tumor cell migration and imparts anti-apoptotic capabilities [143]. In a similar vein, the CXCL12/STAT3 axis has been implicated in research related to brain injury. In a rat model of middle cerebral artery occlusion (MCAO), the administration of oncostatin M (OSM) was found to significantly upregulate CXCL12 in bone marrow-derived mesenchymal stem cells (BMSCs) via the STAT3 and ERK pathways. Moreover, OSM notably enhanced the expression of VEGF and MMP-2. OSM also facilitated the secretion of CXCL12 in astrocytes through the STAT3 and ERK pathways, thereby augmenting BMSC migration [144]. Liang et al. demonstrated that IL-6 activates the Wnt/β-catenin pathway via STAT3/ERK signaling, thereby promoting EMT in colorectal cancer cells [145]. These findings offer valuable insights into the interplay between the CXCL12-mediated STAT3 pathway and the ERK and Wnt/β-catenin pathways, thereby presenting a promising direction for future research.

Cancer stem cells (CSCs) are predominantly responsible for chemotherapy resistance and tumor recurrence. Research has demonstrated that when co-cultured in vitro with human bone marrow-derived mesenchymal stem cells, CD15-induced CSCs (iCSCs) isolated from 293FT cells exhibit characteristics akin to tumor stem cells and proliferate effectively. Subsequent investigations have discovered that iCSCs express the chemokine CXCL12 and its receptor CXCR4, which activate the Fut4 gene via the CXCR4/ERK/ELK-1 signaling pathway, thereby maintaining iCSCs in an undifferentiated state through the CXCR4/AKT/STAT3 signal [90]. Mesenchymal stem cells (MSCs) possess therapeutic potential for the repair of damaged tissues. In studies pertaining to skin wound healing, it has been observed that MSC exosomes activate the AKT, ERK, and STAT3 signaling pathways and induce the expression of CXCL12 [146]. Bone marrow mesenchymal stem cells (BMSCs) demonstrate potential regenerative effects on brain damage, and it has been established that oncostatin M (OSM) influences the proliferation and migration of mesenchymal stem cells.

## 5. Targeted Therapy for the CXCL12/CXCR4/ACKR3 and STAT3 Pathways

Given the deleterious role that the CXCL12/STAT3 axis plays in a variety of tumors and diseases, it is of utmost importance to target this axis for therapeutic intervention in tumors. Pharmacological intervention serves as the cornerstone in the treatment of cancer. Notably, within the realm of tumor metastasis, treatment with vitamin D has been demonstrated to selectively inhibit the expression of p-STAT3, zinc finger E-box binding homeobox 1 (Zeb1), and Vimentin while enhancing the expression of E-Cadherin. Intriguingly, in vivo studies further substantiate that a deficiency in vitamin D results in an upregulation of Zeb1 and p-STAT3 expression in primary breast tumor cells, thereby augmenting the expression of CXCL12 within the pulmonary stroma [147]. Furthermore, in the context of lung metastasis, a deficiency in vitamin D facilitates the co-localization of CXCL12 and its receptor CXCR4 [147].

Pancreatic ductal adenocarcinoma (PDAC), one of the most lethal forms of cancer, is distinguished by the presence of highly tumorigenic and metastatic cancer stem cells (CSCs). Treatment with the antimalarial drug chloroquine in vitro has been observed to significantly diminish CSCs, thereby reducing in vivo tumorigenicity and invasiveness across a wide range of pancreatic cancers [148]. The combination of gemcitabine with in vivo therapy results in more effective tumor eradication and an improved overall survival rate. From a mechanistic perspective, the inhibitory effects of chloroquine are attributed to the suppression of the CXCL12/CXCR4 signaling pathway, leading to a reduction in the phosphorylation of ERK and STAT3 [148]. Furthermore, SKLB-850, a novel SYK inhibitor, has been found to markedly suppress proliferation and induce apoptosis in B-cell lymphomas (BCLs). Further mechanistic studies reveal that SKLB-850 effectively inhibits the activation of the Syk/ERK, Src/FAK, and JAK2/STAT3 pathways, while significantly reducing the secretion of chemokines CCL3, CCL4, and CXCL12 [149].

Traditional Chinese medicine (TCM) plays a pivotal role in modulating the tumor microenvironment (TME), which includes reshaping the immunosuppressive microenvironment, hypoxic conditions, angiogenesis/lymphangiogenesis, and the extracellular matrix [150]. The Qingre Huoxue (QRHX) formula, a 1:1 mixture (w/w) of Paeonia lactiflora (Shaoyao) and Scutellaria baicalensis (Huangqin), has garnered increasing recognition for its anticancer and anti-inflammatory properties [151]. Notably, QRHX inhibits inflammation and the CXCL12/CXCR4/JAK2/STAT3 signaling pathway, thereby modulating tumor-associated macrophages in murine models and suppressing tumor growth [152]. Additionally, thymoquinone (TQ), the major active component of Nigella glandulifera (Huixiangye Heizhongcao), inhibits the growth of multiple myeloma cell lines. Mechanistically, it suppresses CXCL12-mediated actin polymerization and cell proliferation, significantly reduces STAT3 phosphorylation in multiple myeloma cells, and downregulates Bcl-2 and Bcl-XL expression [153]. Vitexin, a glycosylated flavonoid found in various medicinal plants, exhibits diverse pharmacological activities [154]. It downregulates DNA-binding capacity, reduces nuclear translocation of STAT3, and attenuates epidermal growth factor (EGF)-driven STAT3 gene expression. Furthermore, it inhibits the CXCL12-induced invasion of liver cancer cells [155]. Sinomenine, an alkaloid isolated from traditional Chinese herbs, has been successfully used for centuries in treating rheumatoid arthritis [156]. Notably, sinomenine has gained attention for its antitumor potential. It has been demonstrated to inhibit proliferation and induce apoptosis in various human tumor cells [157,158,159,160]. Ye et al. found that sinomenine induces S-phase arrest, inhibits invasion and metastasis in osteosarcoma cells by suppressing the CXCR4-STAT3 pathway, and mitigates bone destruction and angiogenesis mediated by osteoclastogenesis in osteosarcoma [161]. Turmeric (*Curcuma longa*), a perennial herb of the ginger family, has shown anticancer potential in a 7,12-dimethylbenz[a]anthracene (DMBA)-induced carcinogenesis hamster model [162]. Aggarwal et al. discovered that turmeric downregulates CXCR4 expression and inhibits tumor cell proliferation by suppressing the STAT3 pathway [121].

Chimeric antigen receptor T cells (CAR-T) specific to Claudin182 (CLDN18.2) have demonstrated therapeutic efficacy in CLDN18.2-positive pancreatic ductal adenocarcinoma (PDAC). The presence of CXCR4 has been observed to enhance the infiltration of CAR-T cells, thereby enabling CXCR4 CAR-T cells to more abundantly infiltrate tumor sites. Through the STAT3/NF-κB/CXCL12α axis, these cells inhibit the migration of myeloid-derived suppressor cells, thus achieving enhanced therapeutic effects in the treatment of CLDN18.2-positive pancreatic cancer [163]. Synthetic triterpenoids of the oleanane type, including 2-cyano-3,12-dioxooleana-1,9(11)-dien-28-oic acid methyl ester, are emerging as promising pharmaceuticals for the prevention and treatment of breast cancer. These compounds have been shown to inhibit the proliferation of estrogen receptor (ER)-positive breast cancer cells both in vitro and in vivo [164,165,166]. Synthetic triterpenoids have been found to inhibit the infiltration of tumor-associated macrophages into the mammary glands of PyMT mice, reduce the levels of chemokines CXCL12 and CCL2 in primary PyMT mammary tumor cells, and inhibit the phosphorylation of STAT3, thereby suppressing cell proliferation [167].

The CXCL12/CXCR4/ACKR3 axis plays a pivotal role in regulating the migration, dissemination, and homing of leukemia cells. ACKR3 is markedly overexpressed in ALL cells compared to hematopoietic stem cells or normal hematopoietic progenitor cells [168]. MicroRNA-101 (miR-101) has emerged as a novel T-ALL suppressor targeting ACKR3. Exogenous overexpression of miR-101 in leukemia cells inhibited T-ALL cell proliferation and invasion in vitro, and xenograft mouse models demonstrated that miR-101 overexpression suppressed tumor growth and lung metastasis in vivo. Mechanistically, miR-101 suppresses T-ALL tumor development by targeting the CXCL12/ACKR3/STAT3 signaling pathway [86]. T140 is a CXCR4-specific inhibitor. Burger et al. reported that T140 and its analogs inhibited the activity, chemotaxis, and migration of CLL cells in the bone marrow stroma, with the mechanism involving the disruption of CXCL12-induced STAT3 and MAPK phosphorylation [169] (Table 1).

At present, several CXCR4 inhibitors have received FDA approval for clinical use. These include Plerixafor (also known as AMD-3100) [170], Motixafortide [171], and Mavorixafor [172]. Research findings indicate that Plerixafor is capable of inhibiting the activation of the STAT3 signaling pathway, thereby suppressing the development of breast cancer and prostate cancer [52,140]. Furthermore, when Plerixafor is used in combination with triptolide, it reduces the growth of osteosarcoma and inhibits lung metastasis. Notably, the phosphorylation levels of STAT3 decrease in osteosarcoma cells treated with this combination therapy [173]. Additionally, in the context of chronic kidney disease, the CXCR4 inhibitor Plerixafor shows promise as a targeted therapy for renal fibrosis by inhibiting the STAT3 pathway [112]. However, the relationship between Motixafortide and Mavorixafor and the STAT3 pathway remains to be elucidated.

## 6. Discussion

The chemokine CXCL12 and STAT3 signaling pathways play pivotal roles in the development and metastasis of tumors. Therapies targeting tumors, driven by the activation of the STAT3 signaling pathway by CXCL12, warrant considerable attention. A multitude of studies have demonstrated that CXCL12 fosters tumor growth and metastasis, and pharmacological interventions targeting the STAT3 pathway have proven efficacious for various cancers. This review endeavors to elucidate the reciprocal regulatory interaction between CXCL12 and STAT3, thereby addressing the deleterious effects engendered by various tumors.

The CXCL12/CXCR4/ACKR3 axis exhibits an activating interaction with the STAT3 pathway, which contributes to the malignant progression of tumors. However, no study has elucidated the specific molecular mechanisms by which the CXCL12/CXCR4/ACKR3 axis activates the JAK2/STAT3 pathway, and responses to the CXCL12/STAT3 signal transduction may vary across different tumor types and cell lines. The interaction between CXCL12 and its receptors, CXCR4 or ACKR3, plays a crucial role in hematopoietic stem cell proliferation and leukemogenesis. Reviews have highlighted that the CXCL12/CXCR4/ACKR3 axis can activate the STAT3 pathway, leading to leukemia development. Although several drugs (such as Plerixafor, BKT140, and NOX-A12.) targeting CXCL12/CXCR4 have been developed for leukemia treatment, it is essential to recognize that any aberrations (such as intrinsic genetic variations or external factors disrupting homeostasis) may contribute to leukemia initiation. Beyond conferring survival advantages to malignant cells, the activated CXCL12/CXCR4/ACKR3 axis also enhances the invasiveness of hematological malignancies by regulating cell migration and conferring resistance to chemotherapy. Additionally, the potential for leveraging the upregulation of CXCL12 and the subsequent activation of the STAT3 pathway to prevent tumor metastasis warrants further investigation. The present research is largely focused on elucidating the role of CXCL12/STAT3 signaling, yet there is a lack of clear therapeutic strategies. A pivotal issue is how to intervene in the CXCL12/CXCR4/STAT3 signaling pathway to achieve therapeutic effects.

The genesis and progression of tumors are influenced by a myriad of factors, with diverse cytokines and signaling pathways interacting within the tumor. The crosstalk mechanism between CXCL12 and STAT3 varies in different contexts. Initially, CXCL12 was identified as binding with its receptor CXCR4 to influence the STAT3 pathway. However, subsequent to the confirmation of ACKR3 as a receptor of CXCL12, a plethora of studies have verified that the binding of CXCL12 and ACKR3 can also activate the STAT3 pathway, thereby fostering tumor development. Moreover, an abundance of research has also substantiated that the activated STAT3 signaling pathway can promote the secretion of CXCL12 in both stromal cells and tumor cells. Regrettably, no study has yet demonstrated a mutual feedback activation mechanism between CXCL12 and STAT3 in the same cellular context, posing a formidable challenge for future research. Nevertheless, several drug treatments have proven effective against the CXCL12/STAT3 pathway; traditional Chinese medicine, in particular, offers promising prospects. Furthermore, specific targeted intervention of the related proteins in the crosstalk signal axis of CXCL12/STAT3 can pave new avenues for cancer treatment.

## Figures and Tables

**Table 1:** Compendium of therapeutic agents targeting the CXCL12/STAT3 pathway.

Targeting the CXCL12/STAT3 Signaling Pathway	Cancer Type	Mechanisms	Correspondence Author; Year
Qingre Huoxue	Lung cancer	QRHX inhibits inflammation and modulates the tumor-associated macrophages in mice through the regulation of the CXCL12/CXCR4/JAK2/STAT3 signaling pathway.	Jingcheng Dong; 2017 [152]
Vitamin D	Breast cancer	Deficiency in Vitamin D leads to an upregulation of Zeb1 and p-STAT3 expression in primary breast tumor cells, which in turn augments the expression of CXCL12 within the pulmonary stroma.	Richard Kremer; 2021 [147]
Chloroquine	Pancreatic cancer	Chloroquine inhibits the CXCL12/CXCR4 signaling pathway, resulting in reduced phosphorylation of ERK and STAT3, thereby suppressing tumor stem cells.	Christopher Heeschen; 2014 [148]
SKLB-850	B cell lymphoma	SKLB-850 effectively inhibits the activation of the Syk/ERK, Src/FAK, and JAK2/STAT3 pathways, and significantly reduces the secretion of the chemokines CCL3, CCL4, and CXCL12.	Sheng-Yong Yang; 2017 [149]
Thymoquinone	Multiple myeloma	Thymoquinone inhibits actin polymerization and cell proliferation mediated by CXCL12, as well as significantly reduces the phosphorylation of STAT3 in multiple myeloma cells.	Gamal Badr; 2011 [153]
Synthetic triterpenoids	Breast cancer	Synthetic triterpenoid compounds inhibit the expression of the chemokines CXCL12 and CCL2, as well as the phosphorylation of STAT3, thereby suppressing cell proliferation.	Karen Liby; 2012 [167]
CAR-T	Pancreatic cancer	CXCR4 enhances the infiltration of CAR-T cells, and upon tumor entry, CXCR4 CAR-T cells inhibit the migration of myeloid-derived suppressor cells via the STAT3/NF-κB/CXCL12α axis.	Zonghai Li; 2023 [163]
Vitexin	Liver cancer	Inhibition of the STAT3 signaling pathway and suppression of CXCL12-induced invasion of hepatocellular carcinoma cells.	Ahn KS; 2020 [155]
Sinomenine	Osteosarcoma	Inhibition of the CXCR4/STAT3 pathway in osteosarcoma cells induces S-phase arrest and suppresses invasion and metastasis.	Ye ZM; 2016 [161]
Turmeric	Myeloma; Pancreatic cancer; Breast cancer; Colorectal cancer	The compound can downregulate the expression of CXCR4 and inhibit tumor proliferation through the suppression of the STAT3 pathway.	Aggarwal BB; 2013 [121]
MicroRNA-101	leukemia	miR-101 suppresses T-ALL tumor development by targeting the CXCL12/ACKR3/STAT3 signaling pathway.	Yang XY; 2019 [86]
T140	leukemia	T140 effectively inhibits the CXCL12-induced phosphorylation of STAT3 and MAPK, thereby suppressing the activity, chemotaxis, and migration of CLL cells within the bone marrow stroma.	Burger JA; 2005 [169]
